# Do Decision-Making Styles Predict Vagal Control? The Role of Resting Heart Rate Variability

**DOI:** 10.3390/bs14050369

**Published:** 2024-04-28

**Authors:** Adrián Alacreu-Crespo, Raquel Costa, Francisco Molins, Diana Abad-Tortosa, Noemí SanMiguel, Philippe Courtet, Miguel Ángel Serrano

**Affiliations:** 1 Departamento de Psicología y Sociología, Universidad de Zaragoza, 50009 Zaragoza, Spain; aalacreu@unizar.es; 2 Departamento de Psicobiología, Universitat de València, 46022 Valencia, Spain; francisco.molins@uv.es (F.M.); diana.abad@uv.es (D.A.-T.); noemi.miguel@uv.es (N.S.); m.angel.serrano@uv.es (M.Á.S.); 3 Institut National de la Santé et de la Recherche Médicale (INSERM), Neuropsychiatry: Epidemiological and Clinical Research, University of Montpellier, 34090 Montpellier, France; philippe.courtet@umontpellier.fr; 4 Department of Emergency Psychiatry and Acute Care, Lapeyronie Hospital, CHU Montpellier, 34295 Montpellier, France

**Keywords:** decision-making styles, heart rate variability, vagal control

## Abstract

Decision-making styles are a habit-based propensity that drive behavior and affect daily life. *Rational* and *intuitive* decision-making styles have been associated with good mental health. However, the underlying mechanisms are not clear. In the last decade, high basal levels of heart rate variability (HRV) have been proposed as an index of health and emotional control, and this could be one of the variables involved in the effects of decision making on health. Therefore, the aim of this study is to analyze the capability of decision-making styles to predict resting HRV. A cross-sectional study was conducted in a sample of 199 (119 women) young university students, and a resting ECG was recorded to extract frequency domain HRV variables. Subsequently, participants completed sociodemographic data and the General Decision-Making Style questionnaire (GDMS). Results showed that the *intuitive* style predicted high-frequency HRV, while the *avoidant* style predicted less low-frequency HRV. This study presents new data on the relationship between decision-making style and HRV, suggesting that the *intuitive* style has a cardioprotective effect, while the *avoidant* style is related to lower HRV, which has been associated with health vulnerability. In conclusion, this study contributes to the understanding of HRV and its potential as a biomarker for cognitive styles that may improve health.

## 1. Introduction

Human existence is intricately intertwined with the ubiquitous phenomenon of decision making, spanning a continuum from seemingly inconsequential choices to those of profound significance. Scott and Bruce (1995) delineated decision-making styles as patterns of learned behavior, constituting habitual responses shaped by contextual cues that influence perception and action in diverse situational contexts. Based on their research, five distinct decision-making styles have been reported, each delineated through observable behavioral manifestations and not necessarily mutually exclusive in nature. The *rational* style is typified by a methodical and logical approach to problem solving, wherein decisions are guided by systematic analysis and objective information evaluation. In contrast, the *intuitive* style is based on subjective impressions and an acute sensitivity to subtle cues, often privileging feelings, gut instincts, and emotional insights. The *dependent* style is characterized by a propensity to seek external guidance and validation from others, prioritizing input from trusted sources in the decision-making process. Conversely, the *avoidant* style manifests as a deliberate aversion to making decisions whenever feasible, reflecting a preference for avoiding or postponing choice points. Finally, the *spontaneous* style is characterized by a proclivity for impulsive and immediate decision making, wherein choices are made quickly, without consideration of long-term implications [1]. The recognition of these distinct decision-making styles offers valuable insights for understanding and predicting human behavior across various domains, ranging from personal decision-making contexts to organizational settings. As a consequence, the *General Decision*-*Making Styles* questionnaire was developed to assess the different styles. These decision-making styles have been related to daily life decisions and their subsequent consequences [2,3], to coping style [4] in conflict management [5], to goals and motivations [6], and to competence and mental health. Indeed, decision outcomes can have health consequences, and the different styles have been considered risk or protective factors for mental health. That is, the willingness to use *intuitive* style is related to better mental health, to subjective well-being, and to lower levels of stress and depression. Conversely, a predominance of the *avoidant* style is associated with worse mental health and decision-making competency [7], lower self-esteem, impaired self-regulation ability, and difficulty initiating action in decision-making situations [8]. In addition, the *rational* style has been linked to lower perceived stress levels. On the other hand, the *dependent* and *avoidant* style scores are related to sleep problems, and higher perceived stress, cortisol response [9,10], and negative affect [11]. Furthermore, the *avoidant* style has been positively related to depression, anxiety, somatic symptoms, and poor emotional regulation [12]. In fact, individuals exposed to a stressful decision-making task showed a greater amount of negative stress and higher cortisol levels when exhibiting an *avoidant* style [13]. Furthermore, decision-making styles have been studied in suicidal ideation and suicide attempts. It was found that the *spontaneous* style was higher in people with suicide attempts than in people with suicidal ideation. This suggests that the *spontaneous* style may be a discriminator between suicidal ideation and attempts [14]. Overall, these results point out that individual differences in the proneness to certain decision-making styles are related to differential adaptation to changing situations. Thus, the existing literature shows that high scores in *rational* and *intuitive* styles lead to better mental health and, on the contrary, high scores in *dependent*, *avoidant*, and *spontaneous* styles imply maladaptive physiological responses and impairment of mental health.

Decisions, as integral components of daily life, manifest as rapid, flexible, and adaptable responses to situational demands. These cognitive processes necessitate seamless coordination between cerebral cognition and physiological responses within the body [15]. The intricate interplay between inhibitory mechanisms originating from the prefrontal ventromedial cortex (PFCvm) and cardiac parasympathetic (vagal) control leads to a higher heart rate variability (HRV), according to Thayer’s neurovisceral integration model [16] and the polyvagal theory [17]. This vagal control, intricately linked to self-regulation, significantly impacts stress modulation, emotional regulation, cognitive flexibility, and overall well-being [16,17,18]. Therefore, the convergence dynamics of neural and physiological processes underpin decision making.

Moreover, HRV has been proposed as a physiological marker related to flexible responses and behaviors in changing situations [19,20]. Specifically, HRV is a cardiovascular index that reflects the rhythmicity of the heart, and the different rhythms are related to different physiological processes: high-frequency (HF) rhythm is associated with respiratory sinus arrhythmia (RSA) and vagal tone [21] and low-frequency (LF) rhythm with baroreflex function [22]. Basal HRV has been considered an endophenotype [15]; individuals characterized by low basal HRV levels exhibit an augmented vulnerability to mental and cardiovascular illnesses, thereby implicating HRV as a potential biomarker [20]. The interconnection between neural structures allows the prefrontal cortex (PFC) to exert an inhibitory influence, enabling individuals to respond to environmental demands and organize their behavior properly through attention, working memory, or mental flexibility, that is, executive functions. Therefore, HRV represents not only tonic influences of PFC but also a wide range of processes related to adaptation [16]. The literature has demonstrated a relationship between HRV under stress and value-based learning and decision making [23]. Empirical evidence suggests an association between enhanced cognitive performance in decision-making tasks and concurrent patterns of adaptive HRV responses during stressful situations [24] and heightened vagal control both at baseline and across various phases of stress reactivity and recovery [25]. Additionally, day-to-day decisions conform to habits with a cumulative impact on overall health outcomes. These habits are capable of modifying resting HRV; for instance, lifestyle factors such as smoking [26], alcohol consumption [27], or lack of physical exercise [28] are associated with low levels of HRV, indicative of compromised parasympathetic function and heightened susceptibility to adverse health outcomes.

In summary, previous research has pointed out that decision making is associated with basal and reactive HRV [24,25]. In this regard, if the decision-making competences are related to good executive control [29], it is probable, given the relationships between executive control and HRV, that decision-making styles are related to HRV. In the current study, we have focused our interest on decision-making styles as a steady variable, in order to investigate their influence on HRV, in the same way as previous investigations have focused on daily life habits. Therefore, the aim of this study is to investigate whether decision-making styles can predict resting HRV. We hypothesized that greater use of *rational* and *intuitive* styles, which are associated with better mental health, will result in higher HRV; conversely, *dependent*, *avoidant*, and *spontaneous* styles, associated with poor mental health and less control, will predict low HRV.

## 2. Materials and Methods

### 2.1. Participants

The sample was composed of 199 (119 women) Spanish university students, with ages ranging from 18 to 30 (mean ± standard error of mean (SEM) = 21.60 ± 0.17). In order to assure that the sample size was suitable for this investigation, G power was calculated (minimum sample size per condition = 31), based on [25]. Participants were recruited using informative posters and were selected from a larger (*n* = 286) sample using a questionnaire that included the following exclusion criteria, based on Laborde et al.’s (2017) recommendations for performing HRV research: suffering from cardiovascular (*n* = 4), respiratory (*n* = 10), neurological, or psychiatric disease (*n* = 9); drug consumption (*n* = 52); alcohol dependence (*n* = 5); intake of cardioactive medication (*n* = 7) [30].

### 2.2. Procedure

Participants arrived at the laboratory and were informed about the general study procedure. After that, informed consent was obtained from all subjects involved in the study, approved by the Ethics Research Committee of the University of Valencia (ethical approval code H1432733559923). The study was conducted in accordance with the Declaration of Helsinki. Experimental sessions were carried out from 9:00 am to 20:00 pm and lasted around 30 min. Prior to commencing the procedure, the experimenter asked if the participant needed to use the bathroom [31]. Weight, height, and waist-to-hip measurements were taken, and electrodes for electrocardiogram (ECG) assessment were placed. Subsequently, participants were instructed to wait 10 min without any specific instruction on breathing or keeping their eyes open or closed [32]. After that, the ECG electrodes were removed, and participants filled in the General Decision-Making Style questionnaire and sociodemographic data.

### 2.3. Instruments

*Sociodemographic data and questionnaires*

*Sociodemographic data*: An ad hoc questionnaire was used to assess age, socioeconomical variables, and habits, with the objective to control for variables that could influence HRV. Therefore, participants answered questions about sleeping hours the night before the experiment, physical exercise, alcohol consumption 24 h before the experiment, smoking status, and number of cigarettes per day. Finally, the consumption of stimulant beverages, smoking, drinking, and eating two hours before the experiment were controlled for.

*General Decision*-*Making Style* (GDMS): The Spanish version of GDMS [4] was selected, based on the original scale [1]. This questionnaire consists of five sub-scales that measure five decision-making styles—*rational*, *intuitive*, *dependent*, *spontaneous*, and *avoidant*—with good construct validity for the factors. The Cronbach’s alphas for each scale in the present study were as follows: *rational* (α = 0.78), *intuitive* (α = 0.82), *dependent* (α = 0.83), *spontaneous* (α = 0.86), and *avoidant* (α = 0.91). The authors have permission to use this instrument from the copyright holders [4].

*Cardiovascular measures*

The ECG was recorded using three adhesive foam electrodes with conductive hydrogel. The signal was acquired and digitalized at 1000 Hz using PowerLab/16SP hardware (Castle Hill, ADInstruments, Australia) and LabChart software version 5.2. Data were filtered using a 1 Hz low-pass digital filter; after that, the ECG was visually inspected, and abnormal data were edited. Finally, the ECG was analyzed using Kubios Analysis software 2.2 (Biomedical Signal Analysis Group, University of Kuopio, Finland; Tarvanien 2014 [33]). In accordance with Task Force (1996) recommendations for HRV [34], we used the first 5 min of recorded data as a habituation period and analyzed HRV parameters using the last 5 min.

Power spectral analyses of HRV were calculated by means of Fast Fourier Transformation (FFT) to extract frequency domain measures. Spectral power density was expressed in absolute units (ms^2^/Hz). This study computed the very low frequency (VLF) band (between 0.003 and 0.04 Hz); the LF band (between 0.04 and 0.15 Hz), which is an index of the baroreflex function and could be interpreted as both sympathetic and parasympathetic control [35]; and the HF band (between 0.15 and 0.40 Hz), which reflects the RSA and can be taken as an index of parasympathetic control. The total HRV power (HRV_TOT_) was computed by the addition of the frequency bands (HF + LF + VLF). Additionally, we calculated the hertz where HF was collected (HFHz), which is an index of the respiratory rate.

### 2.4. Statistical Analyses

Outliers were calculated using the 3 standard deviations method. One participant was identified as an outlier for HRV variables and, consequently, was removed from the statistical analysis. Kolmogorov–Smirnoff testing was used to check the normality. Age, number of cigarettes, hours of sleep, *rational* style, *intuitive* style, *spontaneous* style, *avoidant* style, VLF, LF, HF, HRV_TOT_, and HFHz did not follow a normal distribution and were normalized with the Log10 method.

In accordance with Laborde et al.’s (2017) [30] recommendations, *t*-tests were conducted to examine the potential influence of confounding variables (eating or drinking stimulant beverages two hours before the experiment, alcohol consumption or strenuous physical activity within 24 h of the experiment) on HRV. To control for the influence of sex on HRV [36], we checked sex differences by means of *t*-tests. Also, Pearson correlations were carried out with HRV variables, decision-making styles, age, body mass index (BMI), waist-to-hip ratio, number of cigarettes per day, and sleep hours. When any of these variables were related to an HRV index, we used this variable as a covariate in the models testing this HRV index.

Second, to test our hypothesis, two-step multiple linear regression analyses were performed to assess whether decision-making styles predicted HRV. For each HRV variable (VLF, LF, HF, and HRV_TOT_), we performed five regressions using each decision-making style (*rational*, *intuitive*, *dependent*, *spontaneous*, and *avoidant*) separately. In the first step, we introduced covariates (i.e., sex, age, BMI, waist-to-hip ratio, eating or drinking stimulant beverages hours before, alcohol consumption, strenuous exercise, number of cigarettes smoked, or hours of sleep) only when these variables showed significant *t*-test results or correlated with the HRV tested variable in the preliminary analyses. In the second step, we introduced the decision-making style scores.

The alpha level was fixed at 0.05. All statistical analyses were performed using SPSS 20.0.

## 3. Results

### 3.1. Preliminary Analyses

Means from demographic characteristics, decision-making styles, and HRV variables separated by sex are shown in Table 1. When comparing men and women, there were significant differences in BMI (*t*_195_ = −2.99, *p* = 0.003, *d* = 0.43), waist-to-hip ratio (*t*_191_ = −9.52, *p* < 0.001, *d* = 1.37), VLF (*t*_196_ = −3.24, *p* < 0.001, *d* = 0.46), LF (*t*_196_ = −4.51, *p* < 0.001, *d* = 0.65), and HRV_TOT_ (*t*_196_ = −2.49, *p* = 0.013, *d* = 0.36); women had lower values for BMI, waist-to-hip ratio, VLF, LF, and HRV_TOT_ than men (Table 1). The rest of the confounding variables checked by the *t*-test did not show significant differences for HRV (*p*’*s* > 0.05).

All the correlations are presented in Table 2. From the confounding variables, HFHz correlated negatively with LF (*r* = −0.23, *p* < 0.001); waist-to-hip ratio correlated positively with VLF (*r* = 0.16, *p* = 0.023); number of cigarettes correlated negatively with VLF (*r* = −0.14, *p* = 0.048) and LF (*r* = −0.14, *p* = 0.048); and hours of sleep before the experiment correlates positively with HF (*r* = 0.17, *p* = 0.016).

### 3.2. Decision-Making Styles Predict HRV

After controlling the confounding variables for each HRV variable, the results indicated that the *intuitive* decision-making style was positively associated with HF (*β* = 0.18, CI_95_: [0.11, 1.17], *r_partial_* = 0.18, *t*(174) = 2.39, *p* = 0.018, *R*^2^ = 0.06) and HRV_TOT_ (*β* = 0.14, CI_95_: [0.01, 0.83], *r_partial_* = 0.14, *t*(195) = 1.99, *p* = 0.048, *R*^2^ = 0.07).

On the other hand, the *avoidant* decision-making style was negatively associated with LF (*β* = −0.13, CI_95_: [−0.49, −0.004], *r_partial_* = −0.14, *t*(190) = −2.00, *p* = 0.047, *R*^2^ = 0.16) and as a trend with HRV_TOT_ (*β* = −0.13, CI_95_: [−0.47, 0.02], *r_partial_* = −0.13, *t*(190) = −1.83, *p* = 0.069, *R*^2^ = 0.06).

*Rational*, *dependent*, and *spontaneous* styles did not have predictive results for any HRV measure (*p’s* > 0.1).

## 4. Discussion

The existing body of literature suggests a positive relationship between better decision-making abilities and enhanced executive control [29]. Hence, the primary objective of this study was to examine the predictive capacity of decision-making styles for resting HRV. On the basis of the associations between executive control and HRV, our hypothesis posited that decision-making styles characterized by greater adaptability would be positively related to higher HRV levels. The findings of this investigation unveiled significant relationships between *intuitive* and *avoidant* decision-making styles and resting HRV, a physiological indicator of emotional regulation, physiological integrity, and mental well-being. Specifically, individuals with higher levels of *intuitive* style exhibited elevated levels of resting HF and HRV_TOT_. Conversely, participants exhibiting pronounced *avoidant* style displayed diminished levels of resting LF and, albeit as a trend, HRV_TOT_. In contrast, no significant associations were observed between the other decision-making styles assessed in this study and resting HRV.

This study revealed that the *intuitive* style was positively related to the HF band during resting conditions, which is a marker of RSA [21], alongside HRV_TOT_. In this sense, heightened HF is indicative of enhanced self-regulation, emotional modulation, and cognitive regulation [15,37,38], underscoring the adaptive nature of *intuitive* decision making in facilitating psychophysiological resilience. Conversely, the findings corroborate the notion that *avoidant* styles exhibit a diminished capacity to adapt to stressful circumstances [13]. Consequently, such tendencies may predispose individuals to long-term health complications and ailments, encompassing disturbances in sleep patterns [9,10], heightened levels of stress, anxiety, or depression [7], negative affective states [11], and compromised mental well-being [12].

In accordance with our hypothesis, the *avoidant* style appears to be associated with diminished HRV in resting conditions, particularly evident within the LF band. Notably, the LF band of HRV serves as a marker of baroreflex function [22], exerting regulatory inhibitory control over heart rate and myocardial contractility [39]. The inhibitory modulation facilitated by baroreflex function confers a cardioprotective effect, thereby rendering lower LF band values indicative of heightened susceptibility to manifesting a reduced capacity for adaptive physiological regulation in response to stressors [20]. Therefore, it is possible that individuals exhibiting an *avoidant* style may be predisposed to the deleterious effects of acute and chronic stress, thereby increasing cardiovascular vulnerability, morbidity, and mortality.

The remaining decision-making styles, the *rational*, *dependent*, and *spontaneous* ones, did not demonstrate significant explanatory power concerning HRV variables. These results agree with a previous investigation [7] that did not find a significant relationship between the former styles and mental health. Research showed the *rational* style was associated with lower stress perception and the *dependent* style with higher stress perception and sleep disturbances [9,10]. One potential rationale for the lack of association between *dependent* and *spontaneous* decision-making styles and HRV could be the inherent characteristics of these styles. The *dependent* style relies on external sources or individuals for decision making, while the *spontaneous* style is characterized by impulsive actions without prior deliberation, suggesting reduced internal cognitive processing compared to other decision-making modalities.

However, this explanation does not correspond with the results for the *rational* style which, in this sense, seems to be driven by more consistent behavior that relates to using more cognitive resources. If we consider that the *rational* style is characterized by the need for a logical understanding of the world, being more closely related to conscientiousness than the rest of the styles [40], people who engage more in this style may exhibit traits such as perfectionism, rigidity, and inflexibility. Although our hypothesis suggested that the *rational* style would explain HRV, our results did not support this relationship. It is important to note that this is the first study to assess cardiovascular parameters in their relation to decision-making styles. This finding suggests that physiological changes associated with decision making may be relatively independent of the tendency to use a *rational* style. Therefore, this study suggests that in environments characterized by variability, challenge, or unpredictability, the most adaptive decision-making approach may be based on feelings [41], a characteristic of the *intuitive* decision-making style. Consequently, habit-driven decision-making processes may lead to a more adaptive behavioral and physiological response to dynamic situational demands. Indeed, prior research has pointed out the pivotal role of effective decision making in eliciting adaptive HRV responses across various contexts, such as in competitive settings [24], and at different stages in a decision-making task [25].

While this research offers valuable insights into the relationship between decision-making styles and HRV parameters, we acknowledge several inherent limitations that warrant consideration. First, the methodological approach adopted, with five linear regression models employed to assess the associations between decision-making styles, HRV parameters, and relevant covariates, raises concerns about the potential for type 2 errors. To minimize this statistical issue, the G power sample size was calculated. Furthermore, the generalizability of our findings may be circumscribed by the specific characteristics of the sample, comprising young and healthy individuals. Consequently, caution must be exercised in extrapolating these results to heterogeneous samples. However, it is noteworthy that our study benefitted from the inclusion of a relatively homogeneous sample, characterized by consistent demographic and health-related attributes.

## 5. Conclusions

In conclusion, the findings of this study underscore the intricate relationship between decision-making styles and HRV as a physiological indicator of mental and physiological well-being. Specifically, our results suggest that individuals exhibiting *intuitive* decision-making styles may experience enhanced cardiac autonomic regulation, as evidenced by higher HRV levels, thereby conferring potential cardioprotective benefits. Conversely, a propensity towards *avoidant* decision-making styles appears to be associated with diminished HRV, which has been implicated in heightened vulnerability to adverse health outcomes. Our investigation did not reveal significant associations between *dependent*, *spontaneous*, and *rational* decision-making styles and HRV. This nuanced differentiation highlights the differential impact of specific decision-making styles on physiological indices of health and underscores the need for interventions centered on promoting healthy decision-making profiles. By elucidating the predictive role of decision-making styles in HRV dynamics, this study contributes novel insights to the literature on psychophysiological interactions. Specifically, our findings suggest that certain decision-making styles may serve as predictors of HRV, thereby offering a potential way of identifying biomarkers of cognitive styles with implications for health outcomes. It is worth noting that our findings have implications for both research and clinical practice. The observed association between *intuitive* decision-making styles and increased basal HRV highlights the multifaceted nature of psychophysiological interactions and the importance of considering cognitive styles in the assessment and management of health outcomes. Moving forward, future research should be advanced to discover and understand the underlying mechanisms driving the observed associations between decision-making styles and HRV, thereby developing targeted interventions aimed at optimizing cognitive functioning and well-being.

## Figures and Tables

**Table 1:** Mean ± SEM of confounding variables, decision-making styles, and HRV parameters by sex (men/women).

Variable	Men	Women	Total
Age	21.82 ± 0.25	21.45 ± 0.22	21.59 ± 0.17
BMI **	24.05 ± 0.35	22.59 ± 0.32	23.17 ± 0.24
Waist-to-hip ratio ***	0.81 ± 0.01	0.73 ± 0.01	0.76 ± 0.004
Number of cigarettes	0.85 ± 0.26	1.44 ± 0.29	1.21 ± 0.20
Hours’ sleep	7.13 ± 0.19	6.76 ± 0.16	6.91 ± 0.12
Rational	4.10 ± 0.07	4.02 ± 0.06	4.06 ± 0.04
Intuitive	3.51 ± 0.09	3.62 ± 0.08	3.58 ± 0.06
Dependent	3.36 ± 0.08	3.57 ± 0.09	3.48 ± 0.06
Spontaneous	2.39 ± 0.09	2.45 ± 0.09	2.43 ± 0.07
Avoidant	2.46 ± 0.11	2.35 ± 0.10	2.39 ± 0.07
VLF ***	148 ± 16	101 ± 8	120 ± 8
LF ***	1776 ± 148	1055 ± 77	1343 ± 79
HF	893 ± 104	852 ± 93	868 ± 69
HRV_TOT_ **	2816 ± 228	2008 ± 148	2330 ± 130
HFHz	0.22 ± 0.01	0.24 ± 0.01	0.23 ± 0.01

** *p* < 0.01, *** *p* < 0.001. Note: BMI = body mass index, VLF = very low frequency, LF = low frequency, HF = high frequency, HRV_TOT_ = total heart rate variability, HFHz = high-frequency hertz.

**Table 2:** Pearson correlations of confounding variables, decision-making styles, and HRV parameters for regression analyses.

	1	2	3	4	5	6	7	8	9	10	11	12	13	14	15
1. VLF	−														
2. LF	0.53 ***	−													
3. HF	0.36 ***	0.62 ***	−												
4. HRV_TOT_	0.55 ***	0.92 ***	0.86 ***	−											
5. HFHz	−0.08	−0.23 *	0.10	−0.10	−										
6. Rational	0.10	0.08	0.001	0.06	−0.06	−									
7. Intuitive	0.03	0.04	0.19 **	0.13	0.02	−0.02	−								
8. Dependent	−0.06	−0.11	0.02	−0.06	0.12	0.19 **	0.01	−							
9. Spontaneous	0.05	0.02	0.09	0.07	0.06	−0.33 ***	0.37 ***	−0.11	−						
10. Avoidant	−0.08	−0.11	−0.06	−0.11	0.07	−0.12	−0.03	0.19 **	0.26 ***	−					
11. Age	−0.02	−0.04	−0.13	−0.08	0.03	0.04	−0.07	−0.02	−0.15 *	−0.17 *	−				
12. BMI	0.12	0.01	0.02	0.02	−0.04	−0.02	−0.03	−0.10	0.01	0.001	0.01	−			
13. Waist-to-hip ratio	0.16 *	0.12	−0.03	0.08	−0.05	0.01	−0.09	−0.14 *	0.001	0.10	0.15 *	0.26 ***	−		
14. Number of cigarettes	−0.14 *	−0.14 *	−0.09	−0.13	0.09	−0.12	0.04	0.16 *	0.09	−0.001	−0.12	0.04	−0.09	−	
15. Hours’ sleep	0.13	0.09	0.17 *	0.14	0.05	0.06	0.01	0.05	−0.02	−0.001	−0.05	−0.11	−0.03	−0.11	

* *p* < 0.05, ** *p* < 0.01, *** *p* < 0.001. Note: VLF = very low frequency, LF = low frequency, HF = high frequency, HRV_TOT_ = total heart rate variability, HFHz = high-frequency hertz, BMI = body mass index.

## Data Availability

The data are available from the corresponding author upon request.

## References

[1] Scott S.G., Bruce R.A. (1995). Decision-Making Style: The Development and Assessment of a New Measure. Educ. Psychol. Meas..

[2] Crossley C.D., Highhouse S. (2005). Relation of Job Search and Choice Process with Subsequent Satisfaction. J. Econ. Psychol..

[3] Gati I., Gadassi R., Mashiah-Cohen R. (2012). Career Decision-Making Profiles vs. Styles: Convergent and Incremental Validity. J. Vocat. Behav..

[4] Alacreu-Crespo A., Fuentes M.C., Abad-Tortosa D., Cano-Lopez I., González E., Serrano M.A. (2019). Spanish Validation of General Decision-Making Style Scale: Sex Invariance, Sex Differences and Relationships with Personality and Coping Styles. Judgm. Decis. Mak..

[5] Loo R. (2000). A Psychometric Evaluation of the General Decision-Making Style Inventory. Pers. Individ. Dif..

[6] Bavolar J., Kacmar P., Lovas L., Durbisova S. (2024). Decision-Making Styles and Goal Striving. J. Behav. Decis. Mak..

[7] Bavol’ár J., Orosová O. (2015). Decision-Making Styles and Their Associations with Decision-Making Competencies and Mental Health. Judgm. Decis. Mak..

[8] Thunholm P. (2004). Decision-Making Style: Habit, Style or Both?. Pers. Individ. Dif..

[9] Allwood C.M., Salo I. (2012). Decision-Making Styles and Stress. Int. J. Stress Manag..

[10] Salo I., Allwood C.M. (2011). Decision-Making Styles, Stress and Gender among Investigators. Policing.

[11] Levin M.E., Krafft J., Pierce B., Potts S. (2018). When Is Experiential Avoidance Harmful in the Moment? Examining Global Experiential Avoidance as a Moderator. J. Behav. Ther. Exp. Psychiatry.

[12] Dworsky C.K.O., Pargament K.I., Wong S., Exline J.J. (2016). Suppressing Spiritual Struggles: The Role of Experiential Avoidance in Mental Health. J. Contextual Behav. Sci..

[13] Thunholm P. (2008). Decision-Making Styles and Physiological Correlates of Negative Stress: Is There a Relation? Cognition and Neurosciences. Scand. J. Psychol..

[14] Qiu T., Klonsky E.D. (2021). Deciding to Die: The Relations of Decision-Making Styles to Suicide Ideation and Attempts. Int. J. Cogn. Ther..

[15] Thayer J.F., Lane R.D. (2009). Claude Bernard and the Heart-Brain Connection: Further Elaboration of a Model of Neurovisceral Integration. Neurosci. Biobehav. Rev..

[16] Thayer J.F., Hansen A.L., Saus-Rose E., Johnsen B.H. (2009). Heart Rate Variability, Prefrontal Neural Function, and Cognitive Performance: The Neurovisceral Integration Perspective on Self-Regulation, Adaptation, and Health. Ann. Behav. Med..

[17] Porges S.W. (2007). The Polyvagal Perspective. Biol. Psychol..

[18] Thayer J.F., Lane R.D. (2000). A Model of Neurovisceral Integration in Emotion Regulation and Dysregulation. J. Affect. Disord..

[19] Laborde S., Raab M., Kinrade N.P. (2014). Is the Ability to Keep Your Mind Sharp under Pressure Reflected in Your Heart? Evidence for the Neurophysiological Bases of Decision Reinvestment. Biol. Psychol..

[20] Thayer J.F., Åhs F., Fredrikson M., Sollers J.J., Wager T.D. (2012). A Meta-Analysis of Heart Rate Variability and Neuroimaging Studies: Implications for Heart Rate Variability as a Marker of Stress and Health. Neurosci. Biobehav. Rev..

[21] Eckberg D.L., Eckberg M.J. (1982). Human Sinus Node Responses to Repetitive, Ramped Carotid Baroreceptor Stimuli. Am. J. Physiol. Heart Circ. Physiol..

[22] De Lartigue G. (2014). Putative Roles of Neuropeptides in Vagal Afferent Signaling. Physiol. Behav..

[23] Forte G., Morelli M., Grässler B., Casagrande M. (2022). Decision Making and Heart Rate Variability: A Systematic Review. Appl. Cogn. Psychol..

[24] Alacreu-Crespo A., Costa R., Abad-Tortosa D., Salvador A., Serrano M.Á. (2018). Good Decision-Making Is Associated with an Adaptive Cardiovascular Response to Social Competitive Stress. Stress.

[25] Forte G., Morelli M., Casagrande M. (2021). Heart Rate Variability and Decision-Making: Autonomic Responses in Making Decisions. Brain Sci..

[26] Sjoberg N., Saint D.A. (2011). A Single 4 Mg Dose of Nicotine Decreases Heart Rate Variability in Healthy Nonsmokers: Implications for Smoking Cessation Programs. Nicotine Tob. Res..

[27] Quintana D.S., McGregor I.S., Guastella A.J., Malhi G.S., Kemp A.H. (2013). A Meta-Analysis on the Impact of Alcohol Dependence on Short-Term Resting-State Heart Rate Variability: Implications for Cardiovascular Risk. Alcohol. Clin. Exp. Res..

[28] Rosenwinkel E.T., Bloomfield D.M., Arwady M.A., Goldsmith R.L. (2001). Exercise and Autonomic Function in Health and Cardiovascular Disease. Cardiol. Clin..

[29] Del Missier F., Mäntylä T., de Bruin W.B. (2012). Decision-Making Competence, Executive Functioning, and General Cognitive Abilities. J. Behav. Decis. Mak..

[30] Laborde S., Mosley E., Thayer J.F. (2017). Heart Rate Variability and Cardiac Vagal Tone in Psychophysiological Research-Recommendations for Experiment Planning, Data Analysis, and Data Reporting. Front. Psychol..

[31] Heathers J.A.J. (2014). Everything Hertz: Methodological Issues in Short-Term Frequency-Domain HRV. Front. Physiol..

[32] Cacioppo J., Tassinary L.G., Berntson G.G. (2007). Handbook of Psychophysiology.

[33] Tarvainen M.P., Niskanen J.P., Lipponen J.A., Ranta-Aho P.O., Karjalainen P.A. (2014). Kubios HRV--heart rate variability analysis software. Comput. Methods Programs Biomed..

[34] Malik M., Camm A.J., Bigger J.T., Breithardt G., Cerutti S., Cohen R.J., Coumel P., Fallen E.L., Kennedy H.L., Kleiger R.E. (1996). Heart Rate Variability. Standards of Measurement, Physiological Interpretation, and Clinical Use. Eur. Heart J..

[35] Berntson G.G., Quigley K.S., Norman G.J., Lozano D.L. (2016). Cardiovascular Psychophysiology. Handbook of Psychophysiology.

[36] Koenig J., Thayer J.F. (2016). Sex Differences in Healthy Human Heart Rate Variability: A Meta-Analysis. Neurosci. Biobehav. Rev..

[37] Duschek S., Muckenthaler M., Werner N., Reyes del Paso G.A. (2009). Relationships between Features of Autonomic Cardiovascular Control and Cognitive Performance. Biol. Psychol..

[38] Zahn D., Adams J., Krohn J., Wenzel M., Mann C.G., Gomille L.K., Jacobi-Scherbening V., Kubiak T. (2016). Heart Rate Variability and Self-Control-A Meta-Analysis. Biol. Psychol..

[39] Shaffer F., McCraty R., Zerr C.L. (2014). A Healthy Heart Is Not a Metronome: An Integrative Review of the Heart’s Anatomy and Heart Rate Variability. Front. Psychol..

[40] Wood N.L., Highhouse S. (2014). Do Self-Reported Decision Styles Relate with Others’ Impressions of Decision Quality?. Pers. Individ. Dif..

[41] Starcke K., Brand M. (2012). Decision Making under Stress: A Selective Review. Neurosci. Biobehav. Rev..

